# Habits and quality of life in portuguese girl adolescents: Association with psychological disturbance distress

**DOI:** 10.1192/j.eurpsy.2021.590

**Published:** 2021-08-13

**Authors:** C. Bento, A.T. Pereira, I. Viega, P. Fonseca, F. Carvalho, A. Macedo

**Affiliations:** 1 University Clinic Of Paediatrics, University of Coimbra/Faculty of Medicine, Coimbra, Portugal; 2 Institute Of Psychological Medicine, Faculty Of Medicine, University of Coimbra, coimbra, Portugal; 3 Institute Of Psychological Medicine, Faculty Of Medicine, University of Coimbra, Coimbra, Portugal

**Keywords:** screen time, quality of life, Portuguese adolescent girls

## Abstract

**Introduction:**

Adolescence is a life period with considerable biological, psychological and social changes. Quality of life is a complex and multifactorial construct that necessarily encompasses the adolescent’s mental well-being. Some life habits like sleep, sport practice and screen time can be either risk factors or protective factors, influencing the quality of life and mental health.

**Objectives:**

To investigate the role of sleep habits, sport practice and screen time in quality of life, and psychological distress in a Portuguese adolescent girl sample.

**Methods:**

409 girls, mean aged 13.51±2.298, from public and private schools in Coimbra answered questions about sleep time, sport practice and screen time habits; the Portuguese versions of the Quality of Life Questionnaire (reduced version) and theAnxiety, Depression and Stress Scale. SPSS 26 was used.

**Results:**

Girls mentioned to sleep 8,2 hours/night during the week and 9,4 hours/night at the weekend. They spend 1,5 hours/day during the week and 1,8 hours/day at the weekend with Screens. They spend 2,1 hours/ week in Sport Practice. Quality of Life was positive correlated with Sleep Time and negative correlated with Screen Time, Anxiety, Depression and Stress (all p<.05). Multiple regression analysis showed that Screen Time, Anxiety and Depression were all significant predictors of Quality of life in girls (p<0,05).

**Conclusions:**

Our findings show that Screen Time is related with poor Quality of Life in girls. These results suggest the importance of addressing Screen Time and Psychological Distress in adolescent girls during adolescent health care consults.

